# Supplementary material for: Enhanced Photodynamic Efficacy Using 1,8-Naphthalimides: Potential Application in Antibacterial Photodynamic Therapy
Source: Molecules. 2022 Sep 6;27(18):5743. doi: 10.3390/molecules27185743 (PMC9504615; doi:10.3390/molecules27185743)
Supplement: Supplementary file 1 [file molecules-27-05743-s001.zip › molecules-1831645-supplementary.pdf]

Supporting Information

Article

Enhanced Photodynamic Efficacy Using 1,8-Naphthalimides: Potential Application in Antibacterial Photodynamic Therapy

Desislava Staneva [1], Awad I. Said [2,3], Evgenia Vasileva-Tonkova [4] and Ivo Grabchev [3,*]

1 Department of Textile, Leader and Fuels, University of Chemical Technology and Metallurgy, 1756 Sofia, Bulgaria

2 Chemistry Department, Faculty of Science, Assiut University, Assiut 71516, Egypt

3 Department of Chemistry and biochemistry, physiology and pathophysiology, Faculty of Medicine, Sofia University “St. Kliment Ohridski”, 1407 Sofia, Bulgaria

4 The Stephan Angeloff Institute of Microbiology, Bulgarian Academy of Sciences, 1113 Sofia, Bulgaria

* Correspondence: i.grabchev@chem.uni-sofia.bg

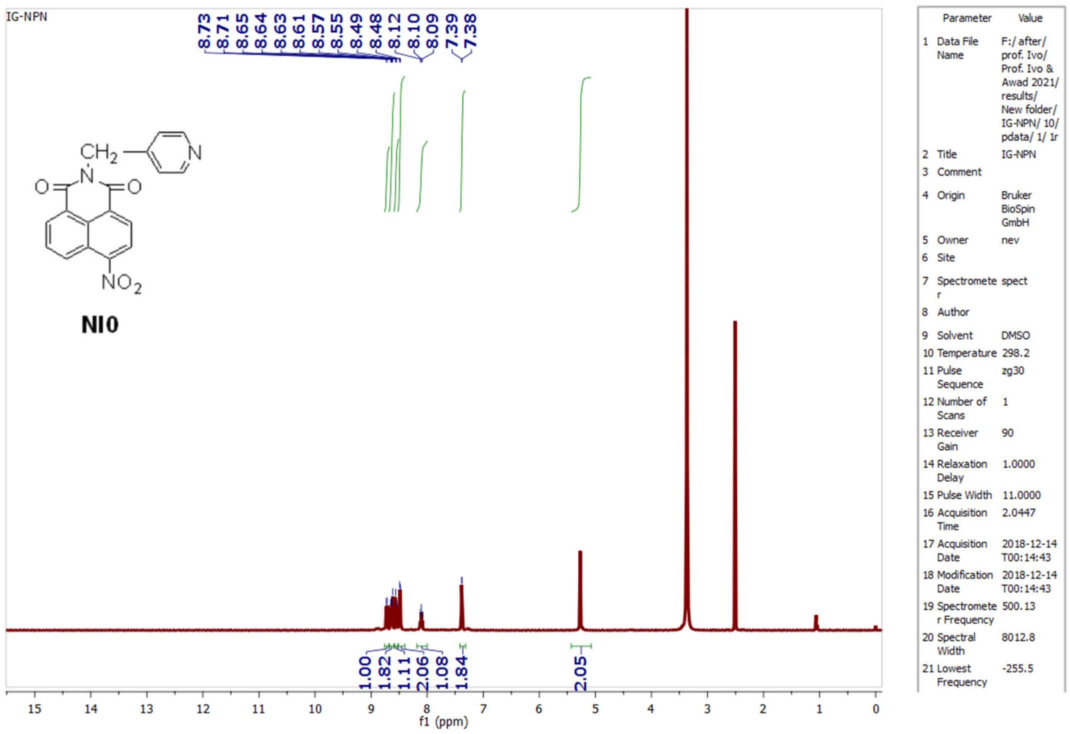

Figure S1. ^{1}H-NMR spectrum of **NI0**.

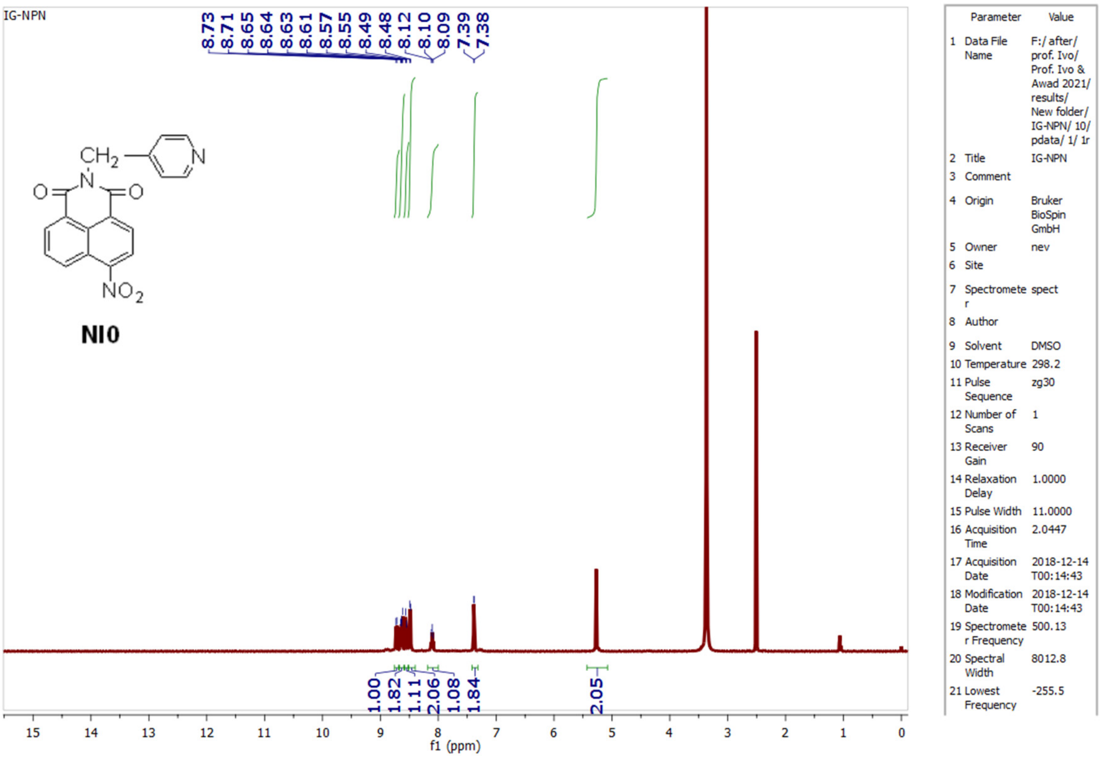

Figure S2. ^{13}C-NMR spectrum of **NI0**.

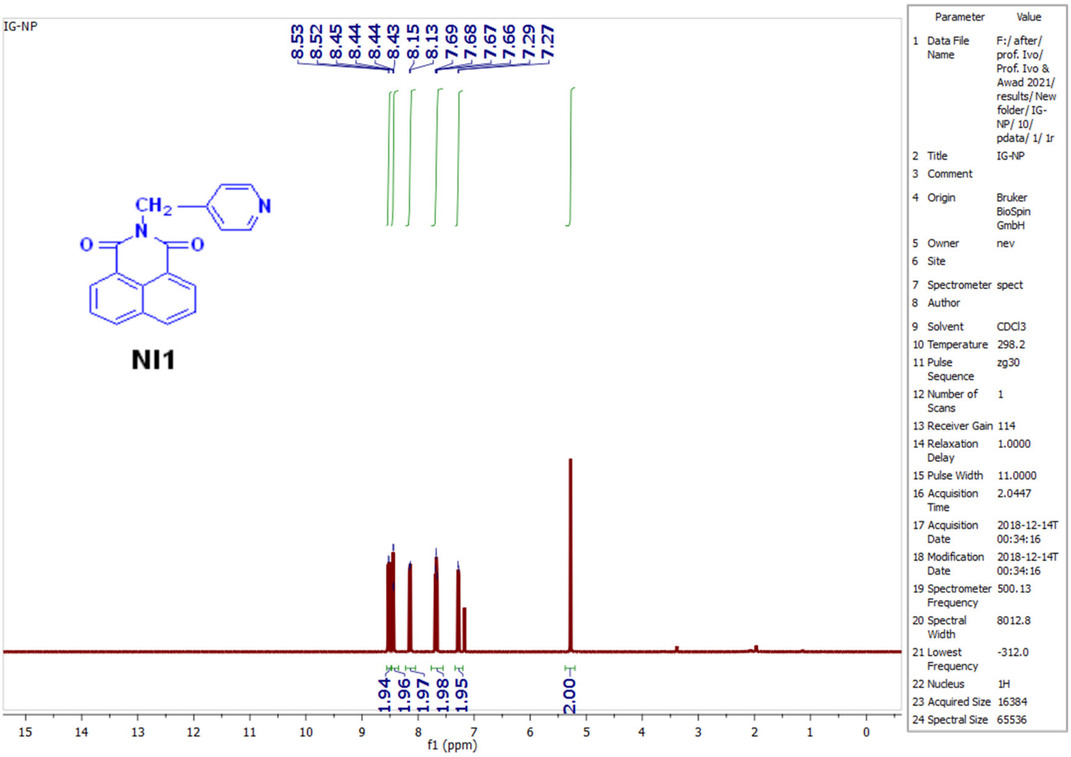

Figure S3. ^{1}H-NMR spectrum of **NI1**.

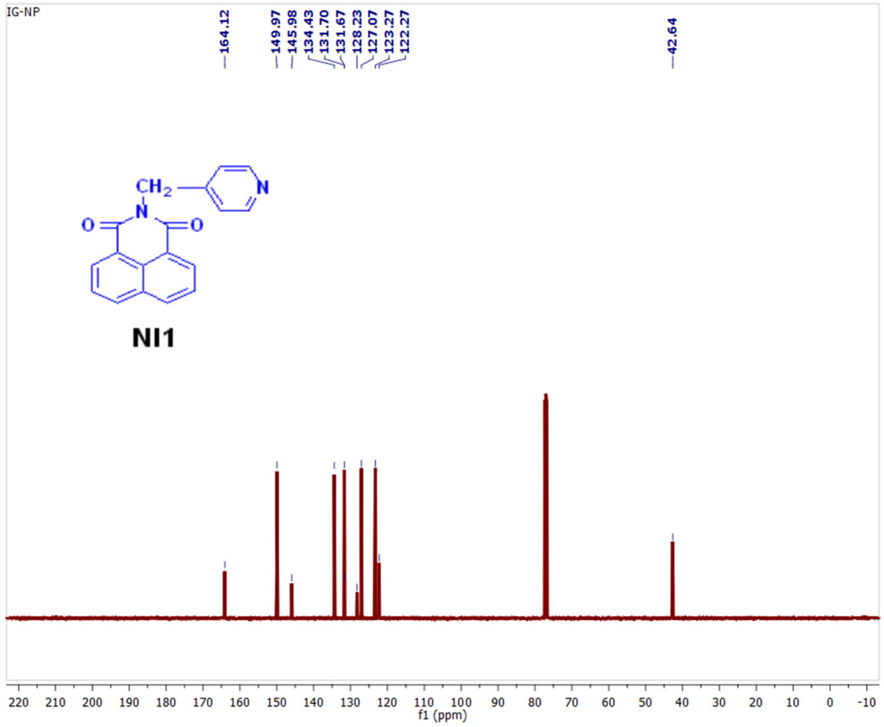

Parameter		Value
1	Data File Name	F:/after/prof. Ivo/Prof. Ivo & Awad 2021/results/New folder/IG-NP/12/pdata/1/1r
2	Title	IG-NP
3	Comment	
4	Origin	Bruker BioSpin GmbH
5	Owner	nev
6	Site	
7	Spectrometer	spect
8	Author	
9	Solvent	CDCl3
10	Temperature	298.1
11	Pulse Sequence	zgdc30
12	Number of Scans	256
13	Receiver Gain	2050
14	Relaxation Delay	2.0000
15	Pulse Width	10.0000
16	Acquisition Time	0.5505
17	Acquisition Date	2018-12-14T00:48:52
18	Modification Date	2018-12-14T00:48:52
19	Spectrometer Frequency	125.76
20	Spectral Width	29761.9
21	Lowest Frequency	-1676.4
22	Nucleus	13C
23	Acquired Size	16384
24	Spectral Size	65536

Figure S4. ^{13}C-NMR spectrum of **NI1**.

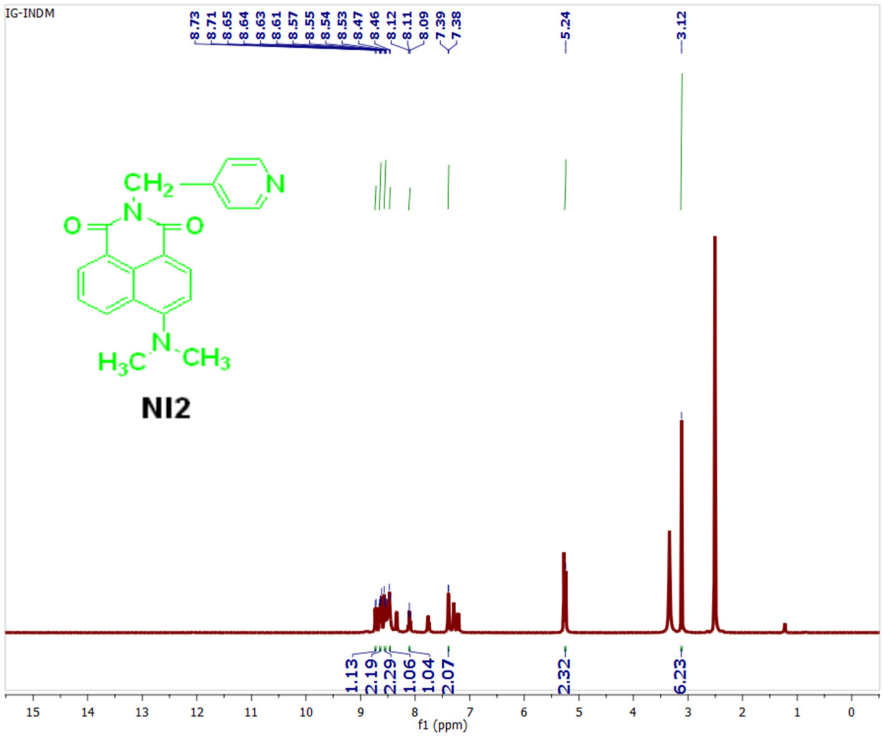

Parameter		Value
1	Data File Name	F:/after/Hungary work/19 20HAS/20HASE/refeences/IG-INDM/10/pdata/1/1r
2	Title	IG-INDM
3	Comment	
4	Origin	Bruker BioSpin GmbH
5	Owner	nmr
6	Site	
7	Spectrometer	spect
8	Author	
9	Solvent	DMSO
10	Temperature	298.2
11	Pulse Sequence	zg30
12	Number of Scans	1
13	Receiver Gain	101
14	Relaxation Delay	5.0000
15	Pulse Width	12.4000
16	Acquisition Time	1.0224
17	Acquisition Date	2022-02-08T14:33:51
18	Modification Date	2022-02-08T14:33:51
19	Spectrometer Frequency	500.13
20	Spectral Width	8012.8
21	Lowest Frequency	-255.4
22	Nucleus	1H
23	Acquired Size	8192
24	Spectral Size	32768

Figure S5. ^{1}H-NMR spectrum of **NI2**.

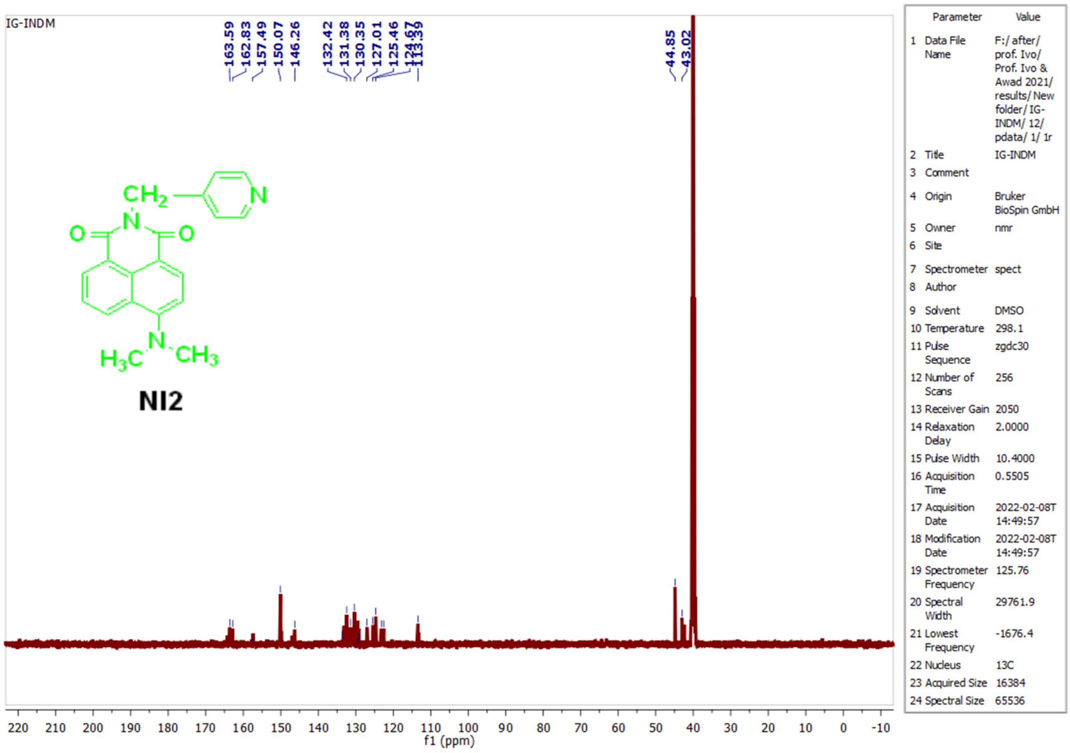

Figure S6. ^{13}C-NMR spectrum of **NI2**.

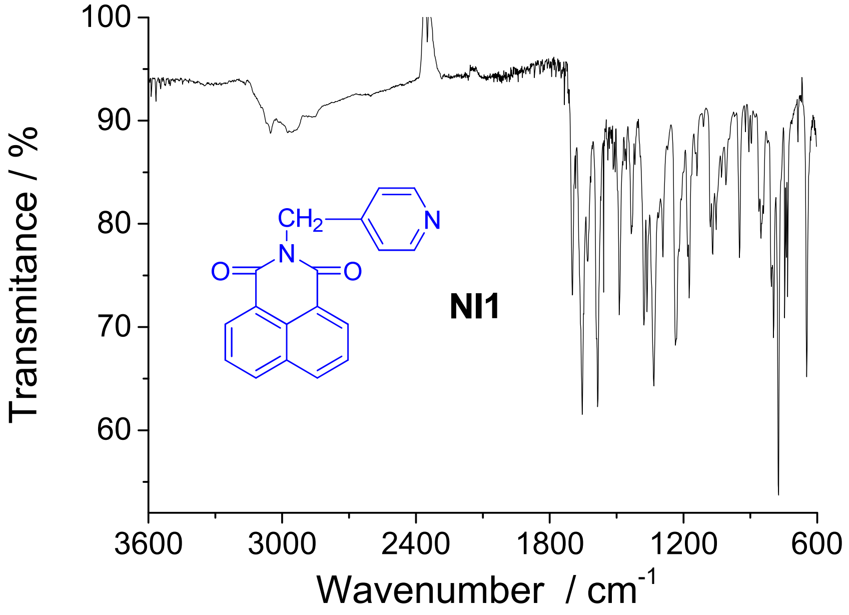

Figure S7. FTIR spectrum of **NI1**.

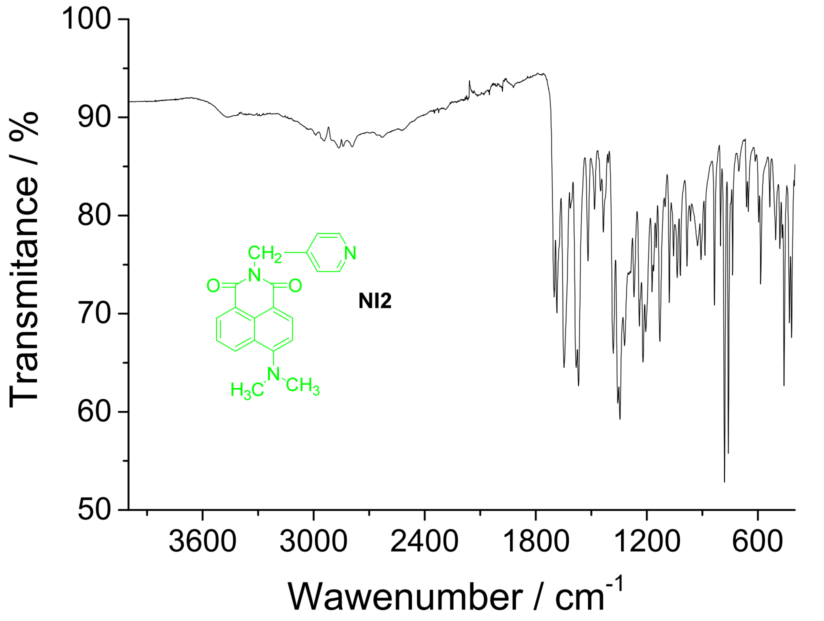

Figure S8. FTIR spectrum of **NI2**.
